# Can role models boost entrepreneurial attitudes?

**DOI:** 10.1504/IJEIM.2017.083476

**Published:** 2017

**Authors:** Katharina Fellnhofer, Kaisu Puumalainen

**Affiliations:** Lappeenranta University of Technology, LUT School of Business and Management, P.O. Box 20, 53851 Lappeenranta, Finland

**Keywords:** entrepreneurship education, entrepreneurial role model, perceived entrepreneurial desirability, perceived entrepreneurial feasibility

## Abstract

This multi-country study used role models to boost perceptions of entrepreneurial feasibility and desirability. The results of a structural equation model based on a sample comprising 426 individuals who were primarily from Austria, Finland and Greece revealed a significant positive influence on perceived entrepreneurial desirability and feasibility. These findings support the argument for embedding entrepreneurial role models in entrepreneurship education courses to promote entrepreneurial activities. This direction is not only relevant for the academic community but also essential for nascent entrepreneurs, policymakers and society at large.

## 1 Introduction

Both Schumpeter’s (1934) as well as Shane and Venkataraman’s (2000) entrepreneurial schools of thought state that entrepreneurship contributes to the overall wealth of our society. Consequently, education that encourages entrepreneurial activities provides an essential ingredient for economic growth (EC, 2013). So, in principle, our wide-ranging knowledge of effective and fruitful pedagogical initiatives are key for our society in the long run.

Research dedicated toward entrepreneurship education (EE) is receiving growing attention from business and political communities and society as a whole (Pittaway and Cope, 2007; Albornoz, 2008; Rasmussen, 2011; Lorz et al., 2013). Still, the latest reviews (e.g. Lorz et al. 2013; Mason and Siqueira 2014) and studies (Rideout and Gray, 2013; Bae et al., 2014) highlight inconsistent research results in prior efforts. Some previous academic works concluded that the EE course has a positive effect on the perceived attractiveness of entrepreneurship (e.g., Peterman and Kennedy, 2003; Fayolle et al., 2006; Souitaris et al., 2007), but other researchers (e.g., Oosterbeek et al., 2010) do not share this point of view and discuss a negative impact. A diverse set of different target groups and different applied pedagogical approaches increase this inconsistency in research results (Mwasalwiba, 2010), and a lack of rigor in research designs and applied research methods (Lorz et al. 2013) appears to contribute to this deficiency.

Apart from developing entrepreneurial knowledge through traditional methods such as business planning within an EE context, creating an entrepreneurial identity is also essential for the entrepreneurial life. The development of an entrepreneurial identity can be enabled through the inspiration of relevant peer groups (Falck et al., 2012; Obschonka et al., 2012). Being confronted with real entrepreneurs’ careers creates a positive awareness (Essers and Benschop, 2007; Clarke, 2011; Wry et al., 2011). However, researchers have not yet illuminated the effects of entrepreneurial role model’s inspiration on individuals’ entrepreneurial attitudes.

Some researchers (Bandura, 1986; Schröder and Schmitt-Rodermund, 2006) argue that observing others can affect an individual’s career choices and decisions. That being so, it is anticipated that entrepreneurial role models will be perceived as encouraging, with a positive attitudinal impact on those considering becoming entrepreneurs. This assumption is based on several earlier studies, which have discussed the existence of role models but have neglected the influence of role model’s inspiration (e.g. Krueger 1993; Matthews and Moser, 1996; Zapkau et al., 2015). Also, based on informal observational education with role models, youth could be encouraged to choose a particular career path (Barling et al., 1998; Krumboltz et al., 1976; Mitchell and Krumboltz, 1984). It seems likely, then, that entrepreneurial role models will affect the perceived desirability and feasibility of an individual becoming an entrepreneur. According to Scherer et al. (1989), the observation of role models enables individuals to learn specific skills, knowledge, and behaviours that are relevant and essential for embarking on a new venture. In particular, earlier findings on human capital (e.g., Holtz-Eakin, 2000; Scherer et al., 1991) indicated that entrepreneurial parents can transfer informal business knowledge to youth. The power of role models can also be illuminated by identification and social learning theory (Gibson, 2004). Identification with role models helps individuals to define their self-concept (Akerlof and Kranton, 2000), and according to social learning theory (Bandura, 1977; 1986), individuals are fascinated by role models who encourage their development (Gibson, 2004). Thus, in this study, the effects of role models for enhancing individual perceived entrepreneurial desirability and feasibility are illuminated. So the following research question represents the driving force in this effort: *Do role models boost entrepreneurial attitudes and how is that accomplished?*

This study is structured as follows. After this introduction, we will outline the theoretical background and develop the hypotheses. Next, we will discuss the research methods and present the results. Finally, we will discuss the practical and theoretical implications and limitations and make additional research recommendations.

## 2 Theoretical background and hypotheses development

Parental role models for entrepreneurs have been intensively discussed (e.g., Chlosta et al., 2012; McDougall et al., 1992), as has the influence of networks (Fernández-Pérez et al., 2016; Lerner et al., 1997) and peer groups (Falck et al., 2012; Giannetti and Simonov, 2009; Koellinger et al., 2007; Nanda and Sørensen, 2010; Stuart and Ding, 2006). Davidsson and Wiklund (1997) reported that the awareness of other entrepreneurs boosts entrepreneurial ambitions. Furthermore, colleagues (Bandura, 1986; Schröder and Schmitt-Rodermund, 2006) argued that observing others can affect an individual’s career choices and decisions. Overall, informal observational education with role models shows potential to encourage one to follow a certain career path (Barling et al., 1998; Krumboltz et al., 1976; Mitchell and Krumboltz, 1984). While these research strands suggest an association between available role models and the choice of entrepreneurship as an attractive career path, in-depth relationships between entrepreneurial role models and entrepreneurial attitudes have not yet been studied.

Perceived entrepreneurial desirability means the perceived attractiveness of becoming an entrepreneur and it is based on Ajzen’s (1991) attitude and subjective norm variables in his Theory of Planned Behaviour (TPB; Krueger et al., 2000). This variable is affected by a social background influenced by the culture, family members, friends and personal entrepreneurial experience. For instance, if receiving a good education for getting well-paid jobs in a large company is communicated in a cultural surrounding, then entrepreneurship will be observed as a less desirable potential career path. If parents are entrepreneurs, in general, entrepreneurship appears to be more attractive for their children than for children of employed parents (Kuehn, 2008; Saeed et al., 2014). In particular, Saeed et al. (2014) stress the significant positive effects of entrepreneurial parental role models concluding that children of entrepreneurs are twice as likely as other children to become self-employed.

Based on Shapero (1975) and, later, Shapero and Sokol’s (1982) intentionality-based process model of the entrepreneurial event, Krueger (1993) found strong support for the idea that entrepreneurial intentions derive from perceived desirability. Similar results have been discussed by later researchers (Guerrero et al., 2008) also in the context of the use of information technology innovations (Moghavvemi and Noor Akma Mohd, 2014). However, Fitzsimmons and Douglas (2011) suggest a typology of nascent entrepreneurs as natural entrepreneurs, accidental entrepreneurs and inevitable entrepreneurs by using a large multi-country sample. Their work might be related to the focal construct considering regional social legitimacy proposed by Kibler et al. (2014). This effort demonstrates and explains how regional social legitimacy influences the perception of the desirability and start-up behaviour. If attitudes are the seed of behaviour to act, then a better view that guides the development of this behaviour becomes crucial. Overall, it seems likely, then, that entrepreneurial role models will affect perceived entrepreneurial desirability to become an entrepreneur. Thus, the following hypothesis is proposed:

H1 Entrepreneurial role models have a positive impact on perceived entrepreneurial desirability.

Perceived entrepreneurial feasibility represents the degree of one’s competence to found a new venture. This awareness is related to Ajzen’s (1991) behavioural control variable because in both an individual assesses the ability to be successful with his or her own business. As a measure of uncertainty, previous entrepreneurial experience and self-confidence in one’s skills and abilities to be successful in managing entrepreneurial tasks are related to this belief in feasibility perceptions (Krueger and Brazeal, 1994; Chen et al., 1998; Krueger et al., 2000; Kuehn, 2008). Overall, this study is in line with previous studies highlighting a positive power of EE on perceived entrepreneurial feasibility (e.g. Peterman and Kennedy, 2003; Fayolle et al., 2006; Souitaris et al., 2007). However, Diaz-Garcia et al. (2015) build on Ajzen’s TPB (1991) to assess the impact of an EE program using a control-group longitudinal design stressing that the candidates perceived greater barriers in the environment over the long run, therefore dropping the feasibility of entrepreneurship as an attractive career path. Nevertheless, this study follows a positive approach suggesting the following:

H2 Entrepreneurial role models have a positive impact on perceived entrepreneurial feasibility.

**Figure 1:**
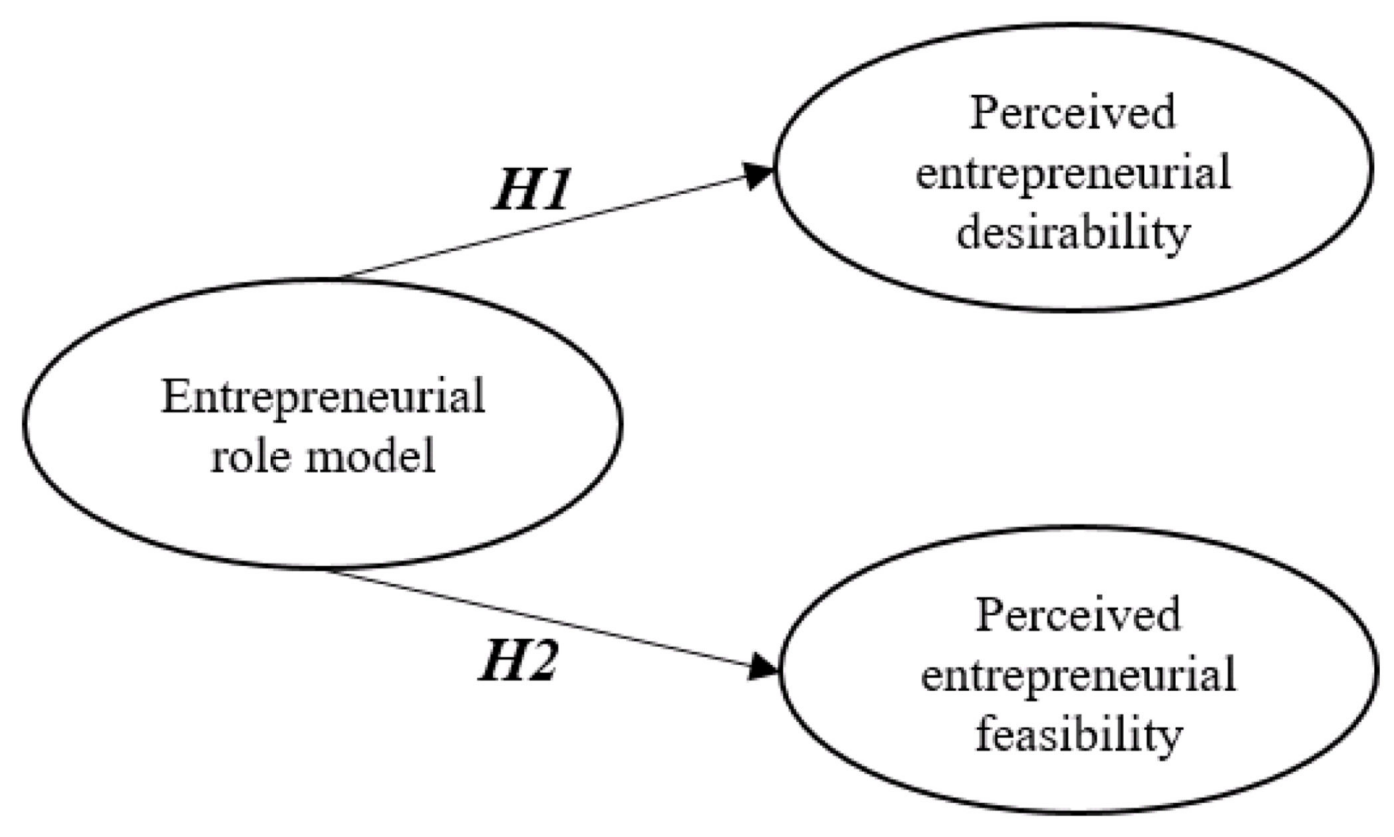
Research model

Figure 1 illustrates the research model.

## 2 Methods

### 2.1 Research data description

The sample consisted of 426 participants including 313 participants aged 18–24 years, with 62.68% male and 37.32% female. The distribution of nationalities was Austria (37.56%), Finland (30.05%) and Greece (24.18%). The data was collected from February 2016 to July 2016 via a questionnaire-based survey online. Table 1 depicts the characteristics of the total sample for this empirical study.

### 2.2 Measurement

In this research study, the risk of common method bias was decreased using the following steps. First, based on the recommendations of Reio (2010) confidentiality and anonymity were guaranteed. Next, this empirical investigation took advantage of well-built scales of past research in EE. The five-item scale for the inspiration of an entrepreneurial role model was modified from Nauta and Kokaly (2001; see Table 5 in the Appendix). Items referring to desirability and feasibility (see Tables 6 and 7 in the Appendix) were taken from Peterman and Kennedy (2003). Participants showed their level of agreement for all items from one indicating strong disagreement to seven indicating strong agreement.

Several reliability and validity tests were implemented following prior recommendations (e.g., Letz and Gerr, 1995; López et al., 2015). Overall, no items had to be omitted. First, a Confirmatory Factor Analysis (CFA) showed an adequate overall model fit (see Table 8 in the Appendix). In this context, all determinants of the correlation matrix of correlating item groups exceeded the threshold of 0.00001, and all communalities were above 0.5. The Kaiser-Meyer-Olkin (KMO) Test for Sampling Adequacy was applied to show whether items transported enough information (Dziuban and Shirkey, 1974) resulting in values all above 0.5 for all items that indicated adequate reliability according to Kaiser (1974). Next, all Cronbach’s alpha values were above 0.784, indicating strong internal consistency (Nunnally, 1978; Hair et al., 1995). Construct validity was significant for all variables (t > 3.1; p < 0.001), as illustrated by the standardised factor loadings (Hair et al., 2010). All indicator reliabilities were also sufficient based on the values recommended by Bagozzi and Baumgartner (1994), and composite reliability was satisfactory showing values above 0.6 (Bagozzi (1988). As specified by Fornell and Larcker (1981), average variance extracted (AVE) was adequate with values above 0.5 for all variables. Finally, the CFA results confirmed the reliability of the research instrument.

Further tests related to construct validity and content validity were performed (Murphy and Davidshofer, 1988; Fraenkel and Wallen, 1993). The results of the bivariate Pearson correlation illustrated in Table 2(a) stressed that all correlations between the variables were significant, confirming that the questionnaire quantified the concept it was intended to measure (Carmines and Zeller, 1990). The variance inflation factor (VIF) was checked for each independent variable. As recommended by Allison (1999) all VIFs were under 2.5. Consequently, multicollinearity was not critical in this work. So the constructs were sufficiently valid and reliable.

### 2.3 Control variables

Control variables such as such as age, gender, nationality and entrepreneurial experience were included in the structural equation modelling (SEM). First of all, Levesque and Minniti (2006) argued that entrepreneurial opportunity costs rise with age. As a consequence, age represented a control variable in our study. Based on the past results of Brush (1992), it was expected that males would tend to be more entrepreneurial. Gender matters when it comes to entrepreneurial initiatives (Fellnhofer et al., 2016). Nationality also represented a controlling variable assuming that the effects in countries in crisis (e.g. Greece) show different effects than in high growth countries (e.g. Finland). Finally, the entrepreneurial experience was also controlled for the assumption that different entrepreneurial experiences effect entrepreneurial perceptions (Fellnhofer and Kraus, 2015).

## 3 Results

Tables 2(a) and 2(b) depict construct means, standard deviations (*SD*) and correlations of the model. All variables correlate significantly.

### 3.1 Goodness of fit of the measurement model

Table 3 confirms the goodness of fit indices for the model. Based on prior studies (e.g. López et al., 2015; Schreiber et al., 2006), the model was tested, and the fit indices are presented in Table 3. Overall, based on the goodness of fit indices, the model can be accepted. The chi-square (X^2^), the goodness of fit index (GFI), comparative fit index (CFI), Tucker-Lewis coefficient (TLI), incremental fit index (IFI) and root mean square error of approximation (RMSEA) exceeded the recommended values. The chi-square value was 656.887, and chi-square/*df* equalled 1.771. The chi/square/*df* is below 5.0, which confirms an adequate fit level according to Hair et al. (2010). Furthermore, the GFI was 0.906 for the model indicating an acceptable fit. In line with prior recommendations (Hu and Bentler, 1999; Byrne, 1994), CFI values were also above the suggested 0.9 level, to be precise 0.924 for the model. The TLI value with 0.911 for the model was above the recommended value of 0.90. The IFI value of 0.926 for the model also indicated an acceptable fit based on recommendations (Bentler and Bonett, 1980; Mulaik et al., 1989). Finally, the RMSEA was 0.043 and so less than 0.07 and, thus, measured as adequate based on MacCallum et al. (1996) and Steiger (2007).

### 3.2 Structural equation modelling

Table 4 presents the parameter estimates for the model. In H1, which proposed that entrepreneurial role models have a positive impact on perceived entrepreneurial desirability, a significant standardised regression weight (SRW) of 0.378 (t = 7.126***, R^2^ = 0.249) was found. In other words, the results indicated that role models significantly and positively affected one’s perceived entrepreneurial desirability. For H2, which proposed that entrepreneurial role models have a positive impact on perceived entrepreneurial feasibility, a less significant SRW of 0.180 was discovered (t = 3.322**, R^2^ = 0.260). In short, the effects of the role models on entrepreneurial desirability were stronger than on entrepreneurial feasibility.

As assumed, entrepreneurial experience significantly and positively influenced the effects of role models on both entrepreneurial desirability (SRW = 0.174, t = 3.328***) and entrepreneurial feasibility (SRW = 0.182, t = 3.331***). Also, there were gender-related effects in both hypotheses. Males tended to perceive both entrepreneurial feasibility (SRW = 0.203, t = 4.005***) and entrepreneurial desirability (SRW = 0.133, t = 2.756***) significantly higher than females. As indicated in Table 2(b) highlighting the gender-related descriptive results, role models were perceived as more important for females than for males even though entrepreneurial desirability and entrepreneurial feasibility were lower for females than for males. This result showed that a role model could play a more important role for females than for males in facilitating entrepreneurial attitudes. Furthermore, Greek participants tended to perceive entrepreneurial feasibility significantly lower than the reference nation Austria (SRW = -0.205, t = –3.539***). Finally, the age group above 56 years perceived entrepreneurial feasibility significantly higher than the other age groups (SRW = 0.214, t = 2.843**), which is in line with the significant results related to the entrepreneurial experience.

## 4 Discussion and conclusions

The primary purpose of this work was to unravel the impact of exposure to entrepreneurial role models on entrepreneurial desirability and feasibility. To this end, a SEM was analysed to explore these effects. The present study builds on previous work such as Kolvereid (1996) and Tkachev and Kolvereid (1999), which developed the TPB [as originally proposed by Ajzen (1991)] further. Additionally, this study draws on social learning theory (Bandura, 1977; 1986) to confirm the significant impact on perceptions when observing entrepreneurial role models. The findings provide empirical support for the significant positive effect of exposure to entrepreneurial role models on both entrepreneurial desirability and feasibility. In general, the results suggest that observation of entrepreneurial role models stimulates entrepreneurial attitudes. In particular, this exposure shows great potential to impact entrepreneurial desirability and feasibility. However, entrepreneurial role model observation alone does not provide students with the necessary knowledge and skills to become a successful entrepreneur but rather serves as an additional ingredient for choosing the career path of an entrepreneur. These findings suggest a need for more engaged role models in EE. In enhancing understanding of the impact of entrepreneurial role models on entrepreneurial desirability and feasibility as potential drivers for nascent entrepreneurs, this study will help in developing further pedagogical instruments to promote entrepreneurial actions and long-term outcomes, with particular regard to sustainability and effective implementation.

### 4.1 Theoretical and practical implications

This contribution has some vital implications. First, it takes an in-depth look at the connection between entrepreneurial role models and entrepreneurial attitudes. Building on Ajzen’s (1991) TPB and Bandura’s (1977, 1986) social learning theory, the study extends current theory by discussing the power of entrepreneurial role models on entrepreneurial desirability and feasibility. Bandura’s (1977) social learning theory postulates that individuals learn from each other via observation, imitation and modelling. Overall, our findings confirm that exposure to entrepreneurial models has a significant positive impact on the entrepreneurial attitudes to start a business. This approach can potentially be exploited on behalf of policymakers, in meeting both the economic requirements and the needs of EE – an idea that has also been raised by Kolvereid and Isaksen (2006).

To empower entrepreneurial behaviour, EE initiatives at different educational institutions should engage role models to stimulate entrepreneurial desirability and feasibility for starting a business. In general, entrepreneurial role model exposure is a means of identifying promising entrepreneurs, and it may be useful for EE initiatives to incorporate entrepreneurial stories with other actions. In line with earlier recommendations (e.g., Van Auken et al., 2006), the present findings confirm that the innovative embedding of role models in EE can have a significant positive effect on those starting a business.

### 4.2 Limitations and future research directions

As with all academic endeavours, this study is subject to certain limitations. First, and most importantly, this study’s sample was comprised of individuals from Austria, Finland and Greece. As a consequence, the findings of this investigation are dependent on cultural and present economic conditions in these countries and may not, therefore, be more widely generalisable. Further economic control variables might be useful to include, not least because Greece was enduring a crisis during the study period. In spite of these limitations, the study serves as a vital point of departure for future research.

The present findings suggest that future research should make further use of TPB-based models. As recommended by Cardon et al. (2013), each of the three domains of passion – inventing, founding and developing – suggest fruitful directions for future research. But a fine-grained examination of these specific objectives was beyond the scope of this study. This study also reiterates Fayolle et al.’s (2014) suggestion that EE research would benefit greatly from longitudinal data on the different factors in entrepreneurial intention, such as exposure to entrepreneurial role models. In this regard, validating all of the TPB variables as significant indirect predictors of entrepreneurial behaviour to entrepreneurial role models would further enrich the potential of the teaching potential of entrepreneurial role models. The present study needs to be repeated with larger samples and in different cultural settings, as well as with different EE target groups. In this regard, it would be interesting to explore how students at the primary level perceive entrepreneurial role models. Finally, given the observed results related to entrepreneurial desirability, future research should place more emphasis on how to increase the desirability of becoming an entrepreneur. This commitment appears crucial in light of the unexplored potential of future entrepreneurs; again, the present study serves as a fruitful point of departure in this regard.

## Supplementary Material

Appendix

## Figures and Tables

**Figure 2:**
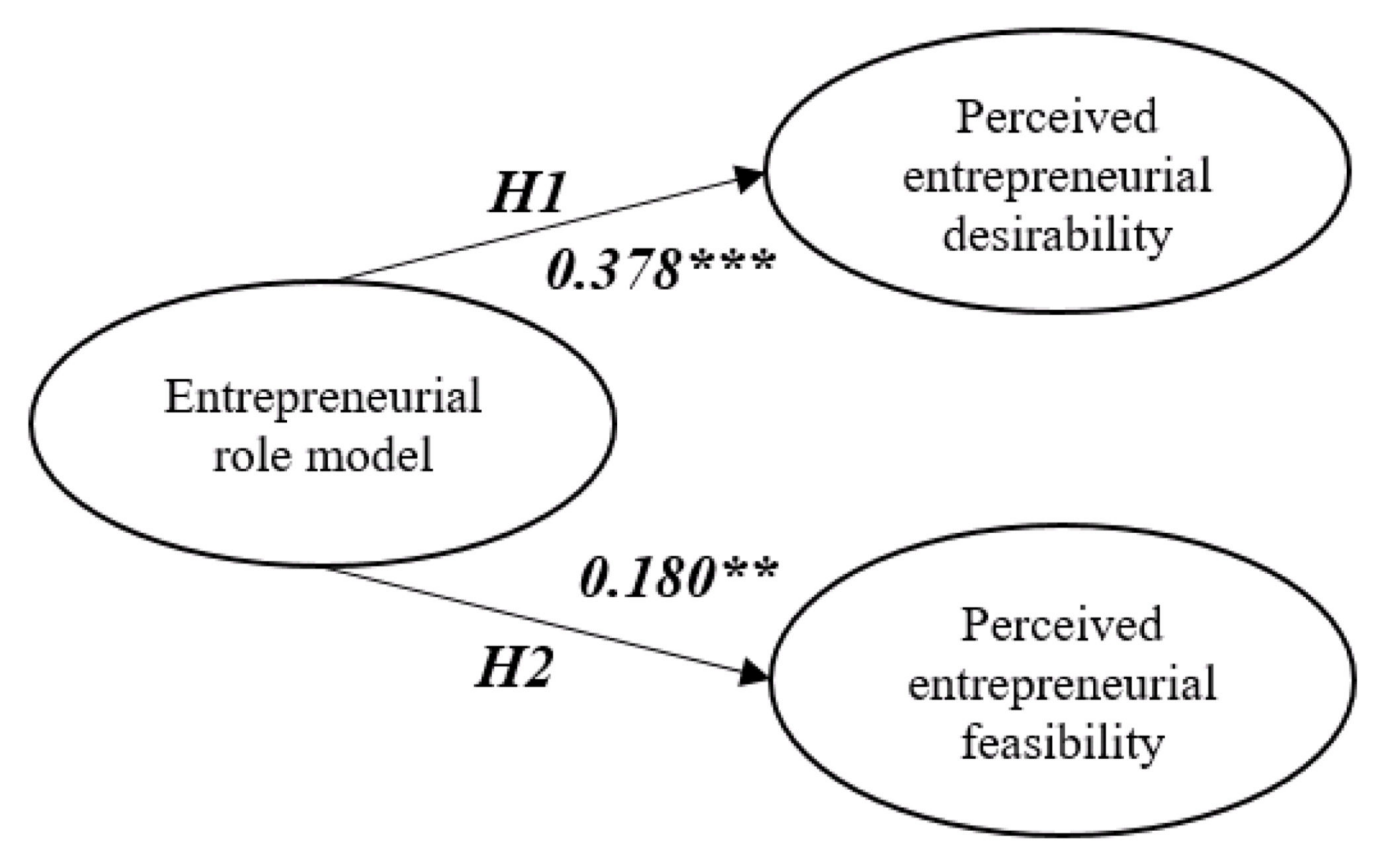
Results of the research models Notes: Standardised regression weights (SRW); significance codes: ***p < 0.001, **p < 0.01.

**Table 1:** Sample

Age	Male	Female	Participants
<18	23	8	31
18–24	187	126	313
25–34	35	16	51
35–44	13	5	18
45–55	7	4	11
56<	2	0	2

	267	159	*426*

*Nationality*	*Male*	*Female*	*Participants*

Austria	126	34	160
Finland	73	55	128
Greece	41	62	103
other	27	8	35

	267	159	*426*

**Table 2(a):** Construct means, *SD*, and pearson correlation (bivariate)

		Mean	*SD*	1	2	3	4
1	Inspiration/modelling	4.02	1.31	1			
2	Desirability	4.47	1.49	.367**	1		
3	Feasibility	3.66	1.16	.226**	.456**	1	
4	Entrepreneurial experience	0.45	0.25	.274**	.266**	.245**	.274**

Notes: n = 426

** correlation is significant at the 0.01 level (two-tailed).

**Table 2(b):** Descriptive results for control variables

		N	Inspiration/modelling		Desirability		Feasibility	
			Mean	*SD*	Mean	*SD*	Mean	*SD*
Gender	Males	267	3.99	1.37	4.58	1.44	3.86	1.19
	Females	159	4.06	1.22	4.29	1.55	3.33	1.03
Nationality	Austria	160	3.97	1.40	4.45	1.55	3.91	1.22
	Finland	128	4.04	1.26	4.31	1.46	3.61	1.06
	Greece	103	4.07	1.22	4.66	1.38	3.25	0.94
Age group	<18	31	3.95	1.40	4.06	1.63	3.82	1.44
	18–24	313	4.03	1.31	4.47	1.45	3.57	1.09
	25–34	51	4.09	1.13	4.67	1.43	3.91	1.23
	35–44	18	3.68	1.60	4.46	1.85	3.77	1.24
	45–55	11	3.93	1.53	5.06	1.51	4.51	1.33
	56<	2	3.90	1.27	3.83	2.59	4.70	2.40

**Table 3:** Summary of goodness of fit indices for the model

Fit indices	X^2^	p-value	Chi-square/df	GFI	CFI	TLI	IFI	RMSEA
Model	656.887	0.00	1.771	0.906	0.924	0.911	0.926	0.043
Recommended values		<0.05	< 5	>0.90	>0.90	>0.90	>0.90	<0.07

Notes: GFI = goodness of fit index, CFI = comparative fit index, TLI = Tucker Lewis index, IFI = incremental fit index, RMSEA = root mean square residual

**Table 4:** Parameter estimates for the model

Parameters standardised (n = 426)	SRW	SE	t-value (p)
*H1: Entrepreneurial role model → Desirability*	*0.378*	*0.062*	7.126***

*Control variables*			
Entrepreneurial experience	0.174	0.329	3.328***
Gender (males, reference females)	0.133	0.157	2.756**
Age (reference <18)			
18–24	0.153	0.289	1.847
25–34	0.094	0.345	1.322
35–44	0.044	0.461	0.737
45–55	0.090	0.528	1.688
56<	0.123	1.654	1.701
Nationality (reference Austria)			
Finland	0.015	0.183	-0283
Greece	0.094	0.200	1.705

*H2: Entrepreneurial role model → Feasibility*	*0.180*	*0.049*	3.322**

*Control variables*			
Entrepreneurial experience	0.182	0.269	3.331***
Gender (males, reference females)	0.203	0.129	4.005***
Age (reference <18)			
18–24	-0.044	0.237	-0.502
25–34	0.015	0.282	0.203
35–44	0.011	0.377	0.185
45–55	0.103	0.431	1.841
56<	0.214	1.352	2.843**
Nationality (reference Austria)			
Finland	−0.037	0.151	–0.667
Greece	−0.205	0.164	−3.539***

Notes: Standard error (SE), standardised regression weights (SRW); significance codes: ***p < 0.001, **p < 0.01.
