# Supplementary material for: Can role models boost entrepreneurial attitudes?
Source: Int J Entrep Innov Manag. Author manuscript; Available in PMC 2017 Apr 26. (PMC5405869; doi:10.1504/IJEIM.2017.083476)
Supplement: Appendix [file NIHMS72278-supplement-Appendix.pdf]

Appendix

Table 5 Entrepreneurial role model – inspiration/modelling

Domain	*Item*	*Mean*	*SD*	*Item total correlation*	*Alpha if item is deleted*
IM_01	There is an entrepreneurial person I am trying to be like in my career pursuits	4.15	1.64	.717	.818
IM_02	There is an entrepreneurial person particularly inspirational to me in my career path	4.15	1.63	.771	.804
IM_03	In the career path I am pursuing, there is an entrepreneurial person I admire	4.25	1.59	.724	.817
IM_04	I have a mentor in my potential entrepreneurial career field	3.36	1.67	.528	.866
IM_05	I know of an entrepreneurial person who has a career I would like to pursue	4.18	1.69	.646	.837

Note: Participants evaluated the statements from 1 (strongly disagree) to 7 (strongly agree)

Source: Modified from Nauta and Kokaly (2001)

Table 6 Perceived entrepreneurial desirability

Domain	*Item*	*Mean*	*SD*	*Item total correlation*	*Alpha if item is deleted*
D_1	I would love to start my own business	4.43	1.76	.804	.784
D_2	I would be very tense to start my own business	4.45	1.56	.685	.889
D_3	I would be very enthusiastic to start my own business	4.55	1.66	.801	.787

Note: Participants evaluated the statements from 1 (strongly disagree) to 7 (strongly agree)

Source: Modified from Peterman and Kennedy (2003)

Table 7 Perceived entrepreneurial feasibility

Domain	*Item*	*Mean*	*SD*	*Item total correlation*	*Alpha if item is deleted*
F_1	It will be easy to start my own business	2.81	1.55	.626	.722
F_2	I will be successful when I have my own business	4.33	1.41	.553	.747
F_3	I won't be overworked when I have my own business	3.31	1.63	.541	.750
F_4	I know enough how to start a business	3.11	1.70	.635	.717
F_5	I am sure about myself	4.77	1.62	.456	.778

Note: Participants evaluated the statements from 1 (strongly disagree) to 7 (strongly agree)

Source: Modified from Peterman and Kennedy (2003)

Table 8 Results of a confirmatory factor analysis (CFA)

Construct	*Items*	*Standardised factor loadings*[a]	*Indicator reliability ≥0.4*[b]	*Composite reliability ≥0.6*[c]	*Cronbach's α ≥0.7*[d]	*AVE ≥0.5*[e]	*Kaiser-Meyer-Olkin measure of sampling adequacy*[f]	*Determinant*[g]	*Bartlett's test of sphericity*[h]
Inspiration/modeling	IM_01	0.829	0.686	0.862	0.859	0.557	0.838	0.094	999.52***
	IM_02	0.887	0.787						
	IM_03	0.782	0.612						
	IM_04	0.518	0.268						
	IM_05	0.655	0.429						
Perceived desirability	D_1	0.888	0.789	0.876	0.875	0.704	0.715	0.191	701.405***
	D_2	0.716	0.512						
	D_3	0.900	0.809						
Perceived feasibility	F_1	0.818	0.670	0.790	0.784	0.438	0.745	0.232	617.773***
	F_2	0.538	0.289						
	F_3	0.579	0.335						
	F_4	0.776	0.602						
	F_5	0.542	0.294						

Notes: [a]All factor loadings are significant ($t > 3.1$; $p < 0.001$).
[b]Bagozzi and Baumgartner (1994).
[c]Bagozzi (1988); Raykov (1997).
[d]All Cronbach's alpha are greater than 0.7 (Nunnally, 1978; Hair et al., 1995).
[e]Fornell and Larcker (1981).
[f]All Kaiser-Meyer-Olkin Measures of Sampling Adequacy are more than 0.5 (Kaiser, 1974).
[g]All determinants of the constructs' correlation matrix are greater than the necessary value of 0.00001.
[h]All significant values conclude that there are suitable correlations in the data set (Bartlett, 1937)
